# Placental expression of estrogen-related receptor gamma is reduced in fetal growth restriction pregnancies and is mediated by hypoxia^†^

**DOI:** 10.1093/biolre/ioac108

**Published:** 2022-05-19

**Authors:** Zhiyong Zou, Lynda K Harris, Karen Forbes, Alexander E P Heazell

**Affiliations:** Maternal and Fetal Health Research Centre, Division of Developmental Biology and Medicine, Faculty of Biology, Medicine and Health, University of Manchester, Manchester, UK; St. Mary’s Hospital, Manchester University NHS Foundation Trust, Manchester Academic Health Science Centre, Manchester, UK; Maternal and Fetal Health Research Centre, Division of Developmental Biology and Medicine, Faculty of Biology, Medicine and Health, University of Manchester, Manchester, UK; St. Mary’s Hospital, Manchester University NHS Foundation Trust, Manchester Academic Health Science Centre, Manchester, UK; Division of Pharmacy and Optometry, Faculty of Biology, Medicine and Health, University of Manchester, Manchester, UK; Maternal and Fetal Health Research Centre, Division of Developmental Biology and Medicine, Faculty of Biology, Medicine and Health, University of Manchester, Manchester, UK; St. Mary’s Hospital, Manchester University NHS Foundation Trust, Manchester Academic Health Science Centre, Manchester, UK; Discovery and Translational Science Department, Leeds Institute of Cardiovascular and Metabolic Medicine, Faculty of Medicine and Health, University of Leeds, Leeds, UK; Maternal and Fetal Health Research Centre, Division of Developmental Biology and Medicine, Faculty of Biology, Medicine and Health, University of Manchester, Manchester, UK; St. Mary’s Hospital, Manchester University NHS Foundation Trust, Manchester Academic Health Science Centre, Manchester, UK

**Keywords:** fetal growth restriction, placental dysfunction, estrogen-related receptor gamma, hypoxia, cell turnover

## Abstract

Fetal growth restriction (FGR) describes a fetus which has not achieved its genetic growth potential; it is closely linked to placental dysfunction and uteroplacental hypoxia. Estrogen-related receptor gamma (*ESRRG*) is regulated by hypoxia and is highly expressed in the placenta. We hypothesized *ESRRG* is a regulator of hypoxia-mediated placental dysfunction in FGR pregnancies. Placentas were collected from women delivering appropriate for gestational age (AGA; *n* = 14) or FGR (*n* = 14) infants. Placental explants (*n* = 15) from uncomplicated pregnancies were cultured for up to 4 days in 21% or 1% O_2_, or with 200 μM cobalt chloride (CoCl_2_), or treated with the *ESRRG* agonists DY131 under different oxygen concentrations. RT-PCR, Western blotting, and immunochemistry were used to assess mRNA and protein levels of *ESRRG* and its localization in placental tissue from FGR or AGA pregnancies, and in cultured placental explants. ESRRG mRNA and protein expression were significantly reduced in FGR placentas, as was mRNA expression of the downstream targets of *ESRRG*, hydroxysteroid 11-beta dehydrogenase 2 (*HSD11B2*), and cytochrome P-450 (*CYP19A1.1*). Hypoxia-inducible factor 1-alpha protein localized to the nuclei of the cytotrophoblasts and stromal cells in the explants exposed to CoCl_2_ or 1% O_2_. Both hypoxia and CoCl_2_ treatment decreased *ESRRG* and its downstream genes’ mRNA expression, but not ESRRG protein expression. DY131 increased the expression of ESRRG signaling pathways and prevented abnormal cell turnover induced by hypoxia. These data show that placental *ESRRG* is hypoxia-sensitive and altered *ESRRG*-mediated signaling may contribute to hypoxia-induced placental dysfunction in FGR. Furthermore, DY131 could be used as a novel therapeutic approach for the treatment of placental dysfunction.

## Teaser

The signaling of estrogen-related receptor gamma (*ESRRG*) is decreased in hypoxia in the cultured explants model and the agonist of *ESRRG*, DY131 can restore hypoxic damages via rescuing the impaired *ESRRG* signals.

## Introduction

Fetal growth restriction (FGR) describes a fetus which does not reach its genetic growth potential; it affects between 5 and 10% of pregnancies. FGR is important as it has both short- and long-term consequences including stillbirth, neonatal death, neurodevelopmental delay in childhood, and increased cardiovascular or metabolic disorders in adulthood [1–3]. The majority of cases of FGR are mediated by abnormal placental structure and function [4]. In normal placentas, terminal villi are covered by syncytiotrophoblast and subjacent cytotrophoblast; the proliferation and fusion of which continuously renews the overlying syncytiotrophoblast to support normal placental function [5]. Reduced remodeling of maternal uterine spiral arteries into low-resistance vessels in early pregnancy is hypothesized to cause poor oxygen (O_2_) delivery to the intervillous space and leads to placental hypoxia. This is thought to contribute to the pathogenesis of FGR, particularly in early-onset and severe cases, which are characterized by aberrant villous trophoblast turnover and placental dysfunction [4, 6, 7]. Indeed, we have previously shown that apoptosis is increased and proliferation is decreased in placental explants cultured in hypoxic conditions (1% O_2_), which is reminiscent of the disordered cell turnover observed in FGR placentas [8, 9].

Estrogen-related receptor gamma (*ESRRG*) is a gene that encodes the orphan nuclear receptor, ESRRG. It is highly expressed in metabolically active organs, including kidney, heart, liver, and placenta [10, 11]. In the human placenta, the ESRRG protein is mainly located in the villous trophoblast, with the highest expression in syncytiotrophoblast [10, 12]. Luo et al. [13] reported that both mRNA and protein expression of ESRRG are increased in placentas from pregnancies complicated by preeclampsia and may be involved in regulating blood pressure homeostasis in mice. Conversely, both mRNA and protein expression of ESRRG were decreased in placentas from pregnancies complicated by FGR [14, 15]. *ESRRG* is also reported to regulate trophoblast proliferation, differentiation, and invasion [12, 15]. siRNA-mediated silencing of ESRRG expression inhibited proliferation and invasion of the trophoblast cell line, HTR-8/SVneo (CVCL_7162) [15], and decreased differentiation of primary cytotrophoblast into syncytiotrophoblast [12]. Based on this evidence, it is plausible that *ESRRG* may play a role in regulating trophoblast function, and dysregulation of *ESRRG* may contribute to the placental dysfunction observed in FGR. Presently, there is no effective treatment of FGR and a better understanding of the pathophysiological mechanism(s) which drive placental dysfunction in FGR placentas, is key for therapeutic advances to be made. Therefore, understanding the mechanisms regulating *ESRRG* in FGR may help to identify new therapeutic targets.

There is some evidence that *ESRRG* expression is regulated by hypoxia, when second-trimester primary cytotrophoblasts were cultured in 2% O_2_, both the mRNA and protein level of ESRRG was decreased and the rate of differentiation into syncytiotrophoblast was reduced [12]. Furthermore, siRNA-mediated knockdown of hypoxia-inducible factor 1-alpha (HIF-1alpha encoded by the gene, *HIF-1A*) induced the expression of *ESRRG* and its downstream gene *CYP191.1* (cytochrome P-450) [12]. Other downstream genes of *ESRRG* in placental cells include hydroxysteroid 17-beta dehydrogenase 1 (*HSD17B1*), placenta-specific protein 1 (*PLAC1*), and hydroxysteroid 11-beta dehydrogenase 2 (*HSD11B2*) [15, 16]. These products are also altered in the placenta in response to hypoxia supporting a role for *ESRRG* signaling in the placental response to hypoxia [17–19].

The *ESRRG* pathway can also be manipulated pharmacologically. GSK5182, a 4-hydroxytamoxifen analog, is a highly selective inverse agonist of *ESRRG*, due to its additional noncovalent interactions with Y326 and N346 at the active site of *ESRRG* [20, 21]. DY131 is an agonist for *ESRRG* which has been used in primary cytotrophoblast and animal models to activate the expression of *ESRRG* [12, 13, 22, 23]. Although past studies have shown reduced expression of *ESRRG* in FGR placentas [14, 15], there is little knowledge as to how dysregulated *ESRRG* signaling is linked to the placental dysfunction observed in FGR [24]. Moreover, the potential for modulation of *ESRRG* pathway has not been explored in the placental explant model. In this study, we hypothesized that the reduced expression of *ESRRG* observed in FGR is mediated by hypoxia, and that agonists of *ESRRG* could rescue hypoxic changes in cultured placental explants.

## Materials and methods

All the reagents were obtained from Sigma (Sigma, UK) unless specified.

### Placental collection

This study was approved by the Research Ethics Committee (08/H1010/55+5) and informed consent was obtained from all participants in Saint Mary’s Hospital, Manchester. All births included in this study were by Caesarean section to eliminate the effects of oxidative stress which occur during normal labor.

In our study, appropriate for gestational age (AGA) (*n* = 14) was defined as an individualized birth weight ratio (IBR) between the 10th to 90th centile and FGR (*n* = 14) was defined as an IBR below the 5th centile. Participant characteristics are shown in Table 1. Women with pregnancy complications such as chronic hypertension, renal disease, preeclampsia, diabetes, gestational diabetes, collagen vascular disease, premature rupture of membranes, and pregnancies complicated with fetal anomalies or chromosomal abnormalities were excluded. Although preeclampsia can also be associated with uteroplacental hypoxia [25, 26], there are additional (and potentially) confounding maternal pathologies involved, thus, placentas from pregnancies complicated by preeclampsia were not included in this study. Term placentas (*n* = 15) from uncomplicated pregnancies were collected for experiments with cultured villous explants (participant characteristics are shown in Table 2). All placentas were collected within 30 min of delivery.

**Table 1:** Participant demographics

Characteristic	AGA controls (*n* = 14)	FGR (*n* = 14)	*P* value
Birthweight (g)	3417.0 ± 625.0 (2916–3930)	2320 ± 795.0 (717–2710)	<0.0001
Birthweight percentile	49.1 ± 18.5 (21.2–79.6)	1.8 ± 1.7 (0–4.5)	<0.0001
Fetal sex (female/male)	8/6	9/5	NS
Gestational age at delivery (weeks)	38.7 ± 0.9 (37.0–40.5)	36.3 ± 2.9 (30.3–39.4)	0.006
Ethnicity			
White British	11 (78.6%)	10 (71.4%)	
Others	3 (21.4%)	4 (28.6%)	
Maternal age (years)	33.9 ± 4.4 (22.0–39.0)	32.3.7 ± 5.0 (20.0–39.0)	NS
Gravidity	2 ± 1 (1–4)	3 ± 1 (2–6)	NS
Parity	1 ± 1 (0–3)	2 ± 1 (0–4)	NS
Nulliparous (%)	5 (35.7%)	2 (14.3%)	0.03
BMI (kg/m^2^)	27.6 ± 5.1 (21––42)	25.9 ± 5.5 (18–36)	NS

Continuous data are shown as mean ± SD or median ± IQR with range, NS, no significant; BMI, body mass index.

**Table 2:** Participant demographics of placental tissue used for cultured explants

Characteristic	Healthy pregnancies (*n* = 15)
Birthweight (g)	3480.4 ± 446.6 (3022–4304)
Birth centile	56.1 ± 29.1 (26.9–98.4)
Fetal sex (female/male)	5/10
Gestational age at delivery (weeks)	39.2 ± 0.3 (39.0–40.0)
Maternal age (years)	33.9 ± 4.8 (25.0–39.0)
Parity	1 ± 1 (0–2)
Gravidity	3 ± 1 (1–4)
Nulliparous pregnant (%)	1 (6.7%)
Ethnicity	
White British	6 (40.0%)
Black African	4 (26.7%)
Pakistani	2 (13.3%)
Croatian	2 (13.3%)
Others	1 (6.7%)
BMI (kg/m^2^)	24.4 ± 4.0 (19.9–28.08)

Continuous data are as mean ± SD or median ± IQR, BMI, body mass index.

### Placental explant culture

Villous explants were prepared as previously described [9]. Villous tissues (*n* = 8) were sampled from three random sites within the placenta, then were further dissected into 2 mm^3^ placental explants, and cultured in 1.5 mL culture medium (10% Connaught Medical Research Laboratories (CMRL)-1066 culture medium (Gibco, UK) supplemented with penicillin (100 IU/mL), streptomycin (100 μg/mL), L-glutamine (100 mg/L), insulin (0.1 mg/L), hydrocortisone (0.1 mg/L), retinol acetate (0.1 mg/L) and 10% fetal calf serum (Gibco, UK). Explants were cultured on Netwells (Corning Inc, NY, USA) at 21% O_2_ (5% CO_2_, 95% air at 37°C) for 24 h then transferred to different conditions for up to 4 days, with media changes every 24 h, in 21% O_2_ (normoxic oxygen levels), 6% O_2_ (physiological oxygen levels), 1% O_2_ or treated with cobalt chloride (CoCl_2_; 200 μM), a drug that mimics hypoxia in vitro by increasing the expression of HIF-1alpha [27]. Hypoxia can stimulate the expression of vascular endothelial growth factor (*VEGF*); therefore, *VEGF* expression was used as a positive control to indicate a hypoxic response in villous explants cultured in 1% O_2_ or with CoCl_2_ [28, 29]. Conditioned culture media was collected at 48, 72, and 96 h; tissue was collected at 96 h and processed for RNA, protein, or immunohistochemistry analysis.

### Treatment with GSK5182 and DY131

Villous explants from uncomplicated pregnancies (*n* = 7) were maintained in 1.5 mL culture medium, composed 1:1 of Dulbecco’s modified Eagle medium (Gibco, UK)/Ham’s F12 (Gibco, UK) supplemented with penicillin (0.6 mg/L), streptomycin (100 μg/mL), L-glutamine (0.292 g/L), and 10% fetal calf serum (Gibco, UK) at 21% O_2_ (5% CO_2_, 95% air at 37°C) for 24 h of culture, as previously described [30]. Culture media was replenished and half of the explants were transferred to the hypoxic incubator (1% O_2_, 5%CO_2_ at 37°C; 24 h), whilst the other half remained at 21% O_2_ for a further 24 h. Media was then replaced with vehicle (0.75% (v/v) Dimethyl Sulfoxide (DMSO)), GSK5182 (20 or 50 μM) or DY131 (20 or 50 μM) and cultured at 21% O_2_ or 1% O_2_ for an additional 48 h without changing culture medium. In each experiment, three villous explants randomly sampled from different sites across the placenta were cultured in the same netwell. Each treatment was replicated in four netwells. The villous explants and conditioned culture medium were harvested at day 4 of culture. At this point, all explants were pooled in one tube, then were randomly selected for RNA extraction, protein extraction or processing for immunohistochemistry.

### RNA extraction and reverse transcription polymerase chain reaction (RT-PCR)

Prior to RNA extraction, villous explants were placed in RNAlater overnight and then stored at −80°C. Cultured villous explants or fresh placental tissue was homogenized (using a handhold homogenizer (SHM1, UK)) and a miRNeasy mini kit (QIAGEN, Germany) was used to extract the total RNA, according to manufacturer’s instructions. An AffinityScript cDNA synthesis kit was used for reverse transcription of mRNA to cDNA, following the manufacturer’s instructions (Agilent Technologies, UK). The PCR primer sequences (Eurofins, UK) for *HIF-1A*, *VEGF*, *ESRRG*, its downstream genes, and *RPLP0* (60S acidic ribosomal protein P0, housekeeping gene) are listed in Supplementary Table 1. Because the mRNA expression of *RPLP0* was stable in human placental tissue [15], it was used as an internal control. The generated cDNA was amplified by PCR by using a powerup SYBR Green (a dsDNA-binding dye) kit (Thermo Fisher Scientific, USA) in the Applied Biosystems Step-one system (Thermo Fisher Scientific, USA). The fold expression was calculated by the 2^−△△CT^ method.

### Protein preparation and western blotting

Protein from villous explants or placental tissue were extracted using Radioimmunoprecipitation assay buffer (RIPA buffer) supplemented with a protease inhibitor complex (1% v/v), phosphatase inhibitor II (1% v/v), and phosphatase inhibitor III (1% v/v). A BCA protein assay (Thermo Fisher Scientific, USA) was used to quantify the protein concentration in the placental lysates.

Around 30 μg of protein from fresh placental tissue supernatants or 120 μg from placental explant supernatants was loaded into a 10% sodium-dodecyl sulfate polyacrylamide gel. The gel was run at 120 V for 80 min and then transferred to a polyvinylidene difluoride (PVDF) membrane (Millipore, Sigma, UK) at 120 V for 80 min. After blocking in 3% (w/v) milk (Marvel, UK) in PBS for 1 h at room temperature, the membrane was incubated with an anti-ESRRG (ab128930, 0.12 μg/mL Abcam, UK) primary antibody at 4°C overnight. Beta-tubulin (ab6046, 0.1 μg/mL Abcam, UK) or Beta-actin (20536-1-AP, 0.09 μg/mL, Proteintech, UK) was used as the housekeeping protein. After washing, the PVDF membrane was incubated with a fluorescent-conjugated secondary antibody (IRDye green, 0.05 μg/mL, LI-COR Bioscience, USA) at room temperature for 1 h. Antibody binding and band intensity was quantified using a Licor Odyssey (LI-COR Bioscience, USA) and Image studio lite software (Image studio lite version 5.2, LI-COR Bioscience, USA). The target protein expression was normalized to house-keeping protein.

### Analysis of human chorionic gonadotropin secretion and lactate dehydrogenase

Human chorionic gonadotropin (hCG) was used as a marker of cytotrophoblast differentiation. hCG in the explant-conditioned culture medium was measured by ELISA (DRG Diagnostics, Marburg, Germany). Villous explants collected at day 4 were lysed in 0.3 M NaOH to provide a value for total protein content, measured using a BioRad protein assay (Bio-Rad Laboratories, Hempstead, UK). hCG secretion was expressed as mIU/mL/h/mg protein. Lactate dehydrogenase (LDH) is an enzyme for conversion of lactate to pyruvate in live cells, it is released from necrotic cells, and is considered a marker for cell viability [31]. LDH levels in explant-conditioned culture medium were measured by a cytotoxicity detection kit (Roche Diagnostics, Mannheim, Germany) based on the manufacturer’s instructions. LDH release was expressed as absorbance units/mg protein/h.

### Immunohistochemical staining

5 μm sections from cultured villous explants or freshly collected placental tissues were transferred onto the slides pre-coated with poly-l-lysine. After dewaxing and dehydration, the slides were treated for antigen retrieval by microwave boiling for 5 min twice at full power (800 W) in 0.01 M citrate buffer (pH 6.0) or Tris/EDTA buffer (pH 9.0) and further incubated with 3% (v/v) hydrogen peroxide for 10 min to block endogenous peroxidase activity. Slides were incubated with non-immune block (10% goat serum and 2% human serum in 0.1% TBST (Tris-Buffered Saline-Tween-20)) for 30 min at room temperature. Sections were incubated with a monoclonal antibody against HIF-1alpha (Abcam, ab51608, 10 μg/mL), ESRRG (Abcam 215947, 10 μg/mL), Ki67 (Dako, 0.174 μg/mL), or M30 (Roche, 0.132 μg/mL) overnight at 4°C. Matched concentrations of isotype-specific non-immune rabbit or mouse IgG were used as a negative control. After washing, biotin-conjugated goat anti-mouse or anti-rabbit antibodies (Dako-Cytomation, 3.85 μg/mL) were applied and incubated for 30 min at room temperature as a secondary antibody. After the incubation with avidin-peroxidase (5 μg/mL) for 30 min, chromogenic substrate diaminobenzidine (DAB; Sigma-Aldrich, UK) was applied to the sections for between 2–10 min. Color development was monitored under a microscope. Harris’s hematoxylin (Sigma-Aldrich, UK) was used to counterstain all slides for 5 min, then was further differentiated with acid alcohol for 2 s. All villous explants and placental tissues were stained in the same batch for comparison; no immunoactivity was observed on the negative controls.

### Statistical analysis

All data are presented as the mean ± standard deviation (SD) (normally distributed) or median ± interquartile range (IQR) (non-normally distributed); a Shapiro–Wilk normality test was used to determine whether the data were normally distributed. GraphPad Prism version 8.0.1 (GraphPad Software, USA) was used to undertake the statistical analysis. Data were assessed using an unpaired *t* test or one-way analysis of variance (ANOVA) for normally distributed data or using a Mann–Whitney *U* test or Kruskal–Wallis, followed by a Friedman multiple comparison test for non-parametric data. QuPath (version 0.2.3, developed by the University of Edinburgh [32]), was used to quantify the immunostaining. DAB staining was quantified to measure the extent of HIF-1alpha, Ki67, and M30 staining; this was expressed as a percentage of the total number of cells. A *P* value <0.05 was considered indicative of statistical significance.

## Results

### Reduced expression of *ESRRG* and its downstream genes in FGR placentas

**Figure 1:**
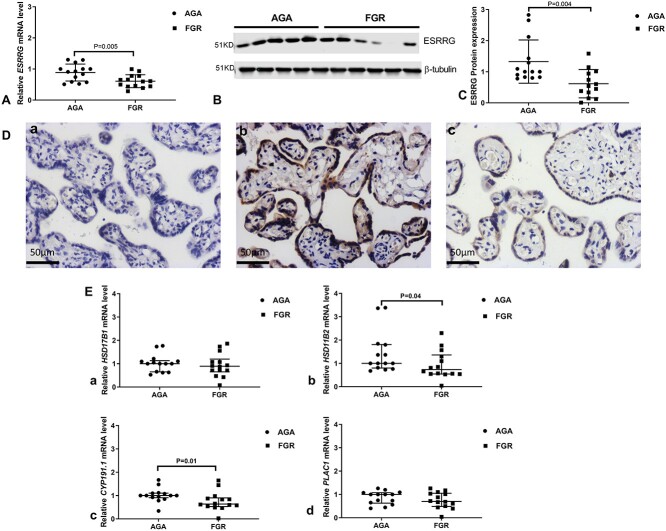
Expression of *ESRRG* and its downstream genes in placentas from AGA pregnancies and those with FGR. (A) mRNA expression of *ESRRG*. (B) Representative Western blot of ESRRG and house-keeping protein beta-tubulin. (C) Quantification of band density. (D) ESRRG protein staining in both AGA (b) and FGR placentas (c); (a) negative control with non-immune rabbit IgG without primary antibody. (E)] the mRNA levels of *ESRRG*’s downstream genes: [a] *HSD17B1*, [b] *HSD11B2*, [c] *CYP191.1*, [d] *PLAC1*. FGR, fetal growth restriction; AGA, appropriate for gestational age; *ESRRG*, estrogen-related receptor gamma; *HSD17B1*, hydroxysteroid 17-beta dehydrogenase 1; *HSD11B2*, hydroxysteroid 11-beta dehydrogenase 2; *CYP191.1*, cytochrome P-450; *PLAC1*, placenta-specific 1. (A) and (C) mean +/− SD, statistical significance was assessed by unpaired *t-*test. (E)] median +/− IQR, Mann–Whitney test.

The mean *ESRRG* mRNA expression level in the FGR group was 34.1% lower than the AGA group (Figure 1A). The mean ESRRG protein level in FGR placentas was reduced, by almost 53.5%, compared with AGA placentas (Figure 1B and C). Immunostaining for protein expression of ESRRG (Figure 1D) in AGA placentas (b) and FGR placentas (c) suggest the expression of ESRRG protein is mainly localized within the syncytiotrophoblast and stromal cells. The mRNA expression level of four genes downstream of *ESRRG*, *HSD17B1*, *HSD11B2*, *CYP19A1.1*, and *PLAC1* were also assessed in FGR and AGA placentas (Figure 1E). The median mRNA level of *HSD17B1* and *PLAC1* was not reduced in FGR compared to that observed in AGA placentas (Figure 1E(a) and (d)); however, the median mRNA levels of *HSD11B2* and *CYP191.1* were significantly decreased by 29 and 25% in FGR placentas, respectively, when compared to AGA placentas (Figure 1E(b) and (c), *P* < 0.05).

### Hypoxia reduces *ESRRG* signaling pathway in cultured villous explants

**Figure 2:**
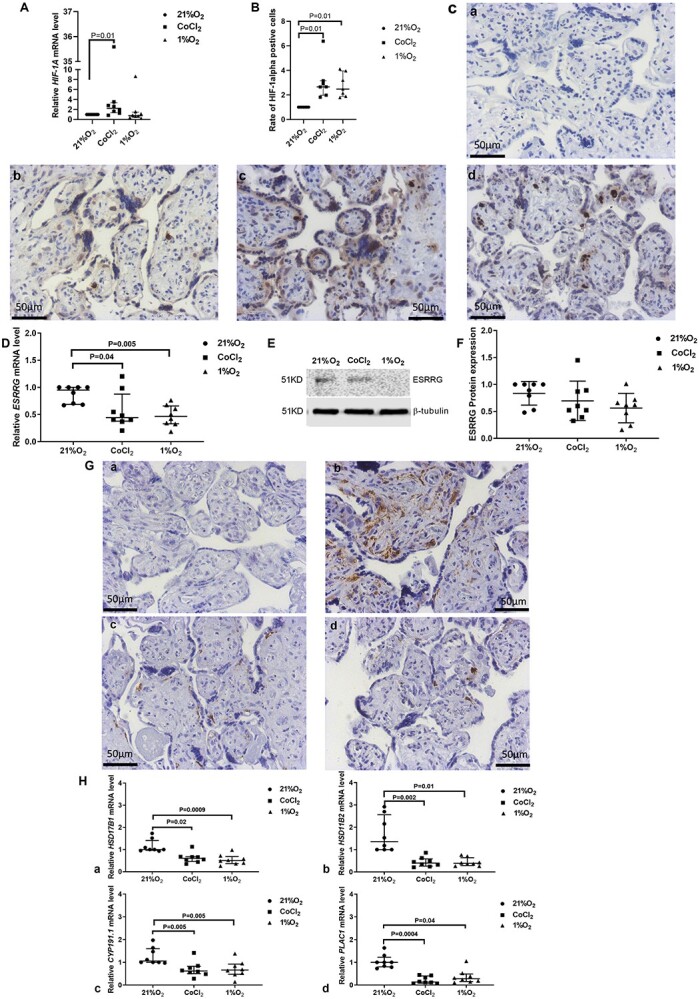
mRNA and protein expression of HIF-1alpha, ESRRG and its downstream genes in explants from placentas cultured in 21% O_2_, 1% O_2_ and following treatment with cobalt chloride (CoCl_2_). (A) mRNA expression of *HIF-1A*. (B) Quantification of HIF-1alpha protein immunostaining. (C) Representative HIF-1alpha protein staining images of explants cultured in 21% O_2_ (b), treated with CoCl_2_ (c), or cultured in 1% O_2_ (d); negative control (non-immune rabbit IgG substituting HIF-1 alpha primary antibody) (a). (D) mRNA levels of *ESRRG*. (E) Representative blot of ESRRG and beta-tubulin (house-keeping gene). (F) Quantification of band density. (G) Representative ESRRG immunostaining images of placental explants cultured under 21% O_2_ (b), CoCl_2_ treatment (c), or 1% O_2_ (d); [a] negative control with non-immune rabbit IgG replacing the primary antibody. Scale bar = 50 μm. [H] mRNA expression of *ESRRG*’s downstream genes: [a] *HSD17B1*, [b] *HSD11B2*, [c] *CYP191.1*, [d] *PLAC1* [A&B], Median +/− IQR; one sample Wilcoxon test. (D, F, and H), Statistical significance was assessed by Friedman test; median +/− IQR.

The mRNA and protein expression and localization of ESRRG was comparable between explants cultured in 21% O_2_ and 6% O_2_ (Supplementary Figure 1A–D). mRNA expression of *ESRRG*’s downstream genes was comparable between explants cultured in 21% O_2_ and 6% O_2_ (Supplementary Figure 1E–H). Compared to the explants cultured in 21% O_2_, mRNA expression of *HIF-1A* was increased in the explants treated with CoCl_2_ (*P* = 0.01), but was unchanged in the explants cultured in 1% O_2_ (Figure 2A). However, the immunostaining results indicated HIF-1alpha protein expression was increased in explants cultured in 1% O_2_ or treated with CoCl_2_ (Figure 2B). HIF-1alpha immunostaining was rarely observed in explants cultured in 21% O_2_ (Figure 2C(b)) but was present in the nuclei of the cytotrophoblasts and stromal cells in explants treated with CoCl_2_ (Figure 2C(c)) or cultured in 1% O_2_ (Figure 2C(d)). *VEGF* mRNA expression was also increased in the explants cultured in 1% O_2_ or treated with CoCl_2_ (Supplementary Figure 2), confirming activation of hypoxia-responsive signaling pathways. Median *ESRRG* mRNA expression was significantly decreased by 51.6% in the explants maintained in 1% O_2_ (Figure 2D) and decreased by 53.7% in placental villous explants exposed to CoCl_2_ (Figure 2D). Western blotting showed that total tissue ESRRG protein expression was unchanged across treatment groups (Figure 2E and F), immunohistochemical staining highlighted that compared to explants cultured at 21% O_2_ (Figure 2G(b)), ESRRG protein expression was reduced in the stroma and trophoblast of explants exposed to CoCl_2_ (Figure 2G(c)) or 1% O_2_ (Figure 2G(d)).

The median level of *HSD17B1* mRNA expression was reduced by 39.1% after exposure to CoCl_2_ (Figure 2H(a)) and by 49.8% after exposure to 1% O_2_ (Figure 2H(a)). Compared to explants cultured at 21% O_2_ the median mRNA level of *HSD11B2* was decreased by three-fold in explants cultured at 1% O_2_ (Figure 2H(b)) or treated with CoCl_2_ (Figure 2H(b)). Moreover, the median mRNA level of *CYP191.1* was decreased by 37.4% after culture in 1% O_2_ (Figure 2H(c)) and by 41.6% in explants exposed to CoCl_2_ (Figure 2H(c)). *PLAC1* mRNA was also reduced in explants exposed to CoCl_2_ (86.5% decrease; Figure 2H(d)) and in explants exposed to 1% O_2_ (72.0% decrease; Figure 2H(d)), compared to those cultured at 21% O_2_.

### GSK5182 and DY131 rescued hypoxia-mediated alterations in *ESRRG* and its downstream genes

**Figure 3:**
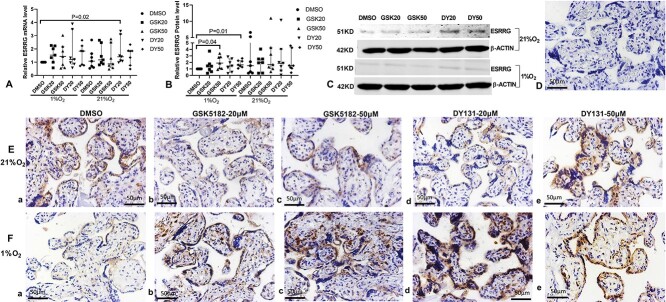
Expression of *ESRRG* in AGA placental explants cultured in 21% O_2_ or 1% O_2_ with the treatment of GSK5182 or DY131. (A) mRNA expression of *ESRRG*; (B) Protein quantification of band density from Western blotting. (C) Representative Western blot of ESRRG and house-keeping gene, beta-actin. (D–F) Representative images of immunochemistry staining for ESRRG in the explants cultured at 21% O_2_ (E(a–e)) or 1% O_2_ (F(a–e)). (D) Negative control with non-immune rabbit IgG. Villous explants were treated with 0.75% DMSO (a), GSK5182 (20 μM) (b), GSK5182 (50 μM) (c), DY131 (20 μM) (d) or DY131 (50 μM) (e). Mark = 50 μM. DMSO, Dimethyl sulfoxide; GSK20, GSK5182 (20 μM); GSK50, GSK5182 (50 μM); DY20, DY131 (20 μM); DY50, DY131 (50 μM). Median +/− IQR; One sample Wilcoxon test was used to examine statistical significance.

**Figure 4:**
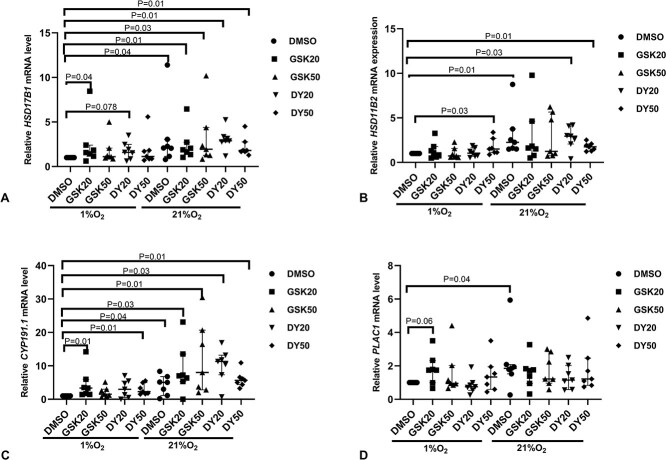
mRNA expression of downstream genes of *ESRRG* in the villous explants with GSK5182 or DY131 treatment in different oxygen concentrations. (A) *HSD17B1* (B) *HSD11B2* (C) *CYP191.1*, (D) *PLAC1.* DMSO, Dimethyl sulfoxide; GSK20, GSK5182 (20 μM); GSK50, GSK5182 (50 μM); DY20, DY131 (20 μM); DY50, DY131 (50 μM). Median ± IQR; One sample Wilcoxon test was used to examine statistical significance.

Compared to the explants cultured in 1% O_2_ with DMSO, treatment of explants cultured at 21% O_2_ with DY131 (20 or 50 μM) increased the mRNA expression of *ESRRG*, *HSD17B1*, *HSD11B2*, and *CYP191.1* (Figures 3A, 4A–C). GSK5182 (20 and 50 μM) increased mRNA expression of *HSD17B1* (Figure 4A) and *CYP191.1* (Figure 4C).

Under hypoxic conditions, GSK5182 and DY131 reversed the low expression of *ESRRG*; GSK5182 and DY131 did not alter *ESRRG* mRNA expression in hypoxia (Figure 3A), but GSK5182 (50 μM) and DY131 (50 μM) increased the protein expression of ESRRG by 1.9-fold and 1.6-fold, respectively (Figure 3B). For *ESRRG*’s downstream genes, compared to the explants cultured in 1% O_2_ DMSO, their mRNA expression was increased in explants cultured in 21% O_2_ DMSO (Figure 4A–D). GSK5182 (20 μM) increased the mRNA expression of *HSD17B1*, *CYP191.1*, and *PLAC1* in 1% O_2_ (Figure 4A, C, and D). Meanwhile, DY131 (50 μM) increased mRNA expression of *HSD11B2* and *CYP191.1* by 1.5-fold and 2.3-fold, respectively (Figure 4B and C).

### DY131 increases the number of cells in cycle and reduces apoptosis induced by hypoxia

**Figure 5:**
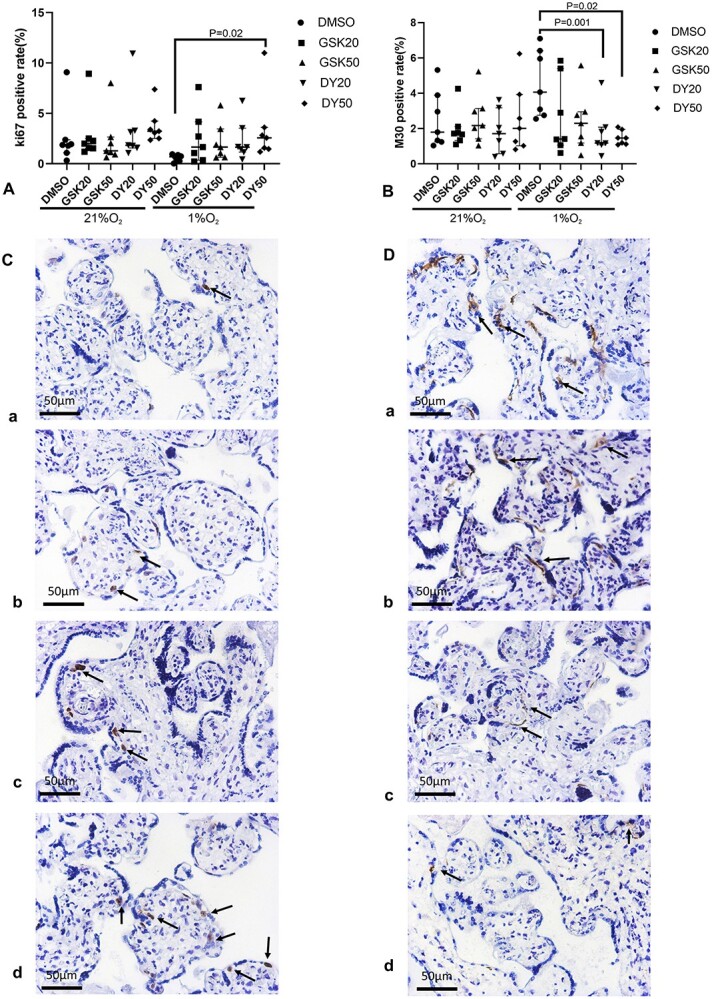
Ki-67 and M30 staining in cultured villous explants following treatment with GSK5182 and DY131. The quantification of Ki-67 (A) and M30 (B) staining. (C) and (D) is the representative images of immunostaining for Ki-67 (C) and M30 (D) in the villous explants cultured at 1% O_2_. [a] 0.75% DMSO; [b] GSK5182 (20 μM); [c] DY131 (20 μM); [d] DY131 (50 μM). Median +/− IQR; Friedman test.

There was no difference in the percentage of cells in cycle, or apoptotic cells, between the explants cultured at 21% O_2_ and 6% O_2_ (Supplementary Figure 3A and B). Compared to villous explants cultured in 21% O_2_, the number of cells in cycle was significantly decreased, and apoptotic cells were significantly increased in villous explants cultured in 1% O_2_ or treated with CoCl_2_ (Supplementary Figure 4A and B). Application of DY131 (50 μM) increased the number of cells in cycle in hypoxic conditions (Figure 5A, and Supplementary Figure 5), and hypoxia-induced apoptotic cell death was significantly decreased in villous explants treated with DY131 (20 and 50 μM) (Figure 5B and Supplementary Figure 6).

### DY131 reduces hypoxia-induced necrosis of villous explants

**Figure 6:**
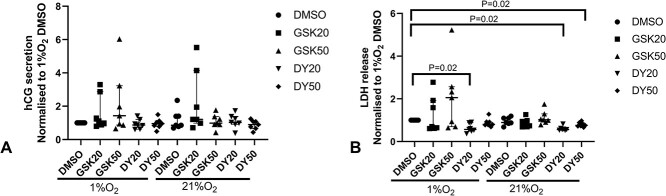
Human chorionic gonadotropin secretion and LDH release in conditioned culture media. (A) The secretion of total hCG. (B) The LDH levels in the culture medium. LDH, lactate dehydrogenase; hCG, human chorionic gonadotropin. Median ± IQR; One sample Wilcoxon’s test was used to examine statistical significance.

Compared to explants cultured in 21% O_2_, hCG secretion was slightly decreased in the explants cultured in 6% O_2_ (Supplementary Figure 7), and LDH levels were similar between the 21% O_2_ group and 6% O_2_ group (Supplementary Figure 7). The mean hCG levels at day 4 of culture were 55.3% or 50% lower following exposure to 1% O_2_ or treatment with CoCl_2_ (Supplementary Figure 8A and B). Compared to the explants cultured in 1% O_2_ with DMSO, neither GSK5182 nor DY131 altered hCG secretion (Figure 6A).

From day 2 to day 4 of culture, there was limited LDH release from the explants cultured in 21% O_2_ (Supplementary Figure 8C); however, at day 4 of culture in 1% O_2_, the mean LDH release was modestly increased (Supplementary Figure 8D) showing a reduction in tissue integrity in this group. This increase in LDH levels normalized in tissue treated with DY131 (20 μM) at 1% O_2_ (Figure 6B). A reduction in LDH levels was also seen in explants cultured at 21% O_2_ that were treated with DY131 (Figure 6B).

## Discussion

**Figure 7:**
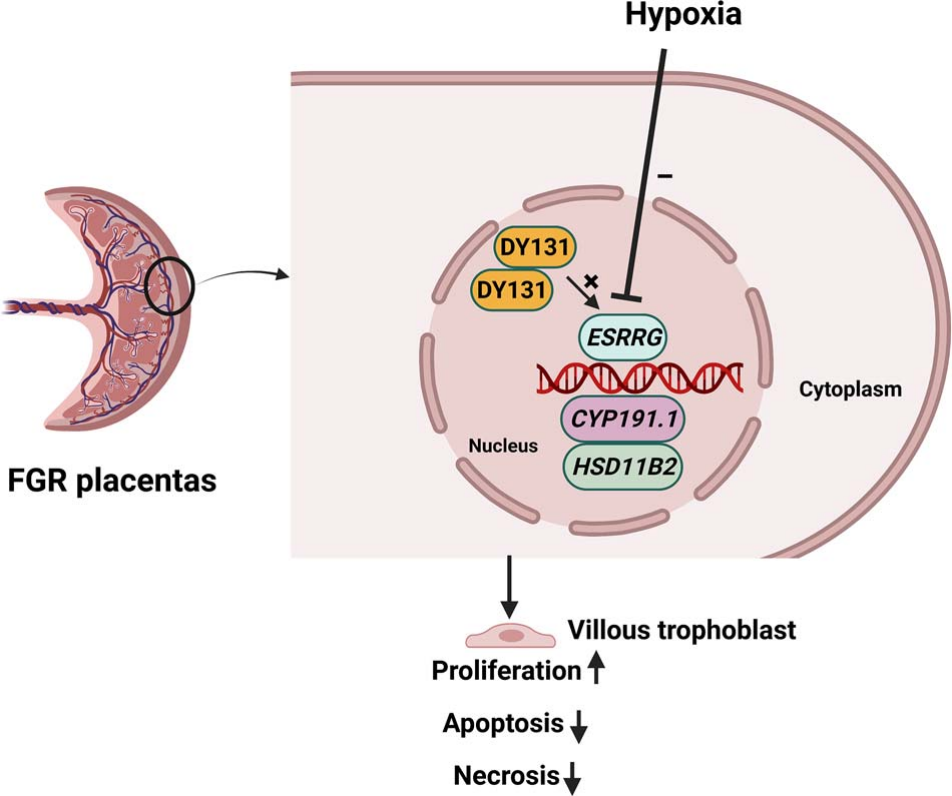
Schematic of the *ESRRG* pathway. *ESRRG*, estrogen-related receptor gamma; *CYP191.1*, cytochrome P-450; *HSD11B2*, hydroxysteroid 11-beta dehydrogenase 2.

This study shows that both mRNA and protein expression of ESRRG and the mRNA expression of its downstream genes are decreased in placentas from pregnancies complicated by FGR, and this can be reproduced by culturing healthy placental explants in 1% O_2_ or exposing them to CoCl_2_. An agonist of *ESRRG*, DY131, rescued the abnormal cell turnover induced by hypoxia, by modulating *ESRRG* signaling. These findings suggest that the *ESRRG* pathway is dysregulated in FGR and may mediate some of the downstream effects of placental hypoxia, a key contributor to placental dysfunction (Figure 7).

### Reduced *ESRRG* signaling in human placentas is related to FGR

Our assessment of AGA and FGR placentas confirms that the mRNA and protein level of *ESRRG* is reduced in FGR placentas, which is consistent with previous studies [14, 15]. These studies used placental samples from southern Chinese and French populations which defined FGR as estimated fetal weight or birth weight less than the 10^th^ percentile. Most of our samples were from Caucasians in the UK, and a more stringent definition of FGR (IBR less than 5th percentile) was applied. The consistent nature of this finding in three different populations suggests that a reduction in *ESRRG* expression is observed in FGR.

To further investigate the *ESRRG* signaling pathway, we quantified the expression of four genes downstream of *ESRRG*. We found the mRNA expression of *HSD11B2* and *CYP19A1.1* were significantly decreased in FGR placentas. *CYP191.1* encodes the aromatase P450 which plays a role in the conversion of C19 steroid precursors into estrogen. Data regarding *CYP191.1* expression in FGR placentas are inconsistent. One previous study of FGR placentas (IBR below 10th centile) found increased expression of *CYP191.1* [33]. It is possible that the reduced expression level of *CYP19A1.1* observed in our study might be related to the severity of FGR. *HSD11B2* encodes an enzyme related to the conversion of active cortisol to inactive cortisone, which is expressed in villous syncytiotrophoblast; placental *HSD11B2* level is correlated with fetal weight and postnatal growth velocity [34–36]. Similar to a previous study [36], our results also revealed reduced mRNA expression of *HSD11B2* in FGR placentas.

*HSD17B1* encodes a steroidogenic enzyme responsible for converting estrone to 17 beta-oestradiol. Previous reports have found that *HSD17B1* is decreased in FGR placentas (IBR less than 10th centile) and activated by *ESRRG* in the HTR-8/SVneo cell line [15]. *PLAC1* was mainly expressed in syncytiotrophoblast and increased in FGR placentas. However, our results did not show a statistical difference in the mRNA expression of *HSD17B1* and *PLAC1* in FGR placentas and this may be due to the difference in race and the definition of FGR placentas among these studies.

### The *ESRRG* signaling pathway is hypoxia-responsive

We used a four-day explant culture at 1% O_2_ or treatment with CoCl_2_ to mimic the placental hypoxia observed in FGR. This model has previously been well characterized and reproduces aspects of FGR, including reduced trophoblast proliferation and increased apoptosis [8, 9, 37]. Both hypoxia and CoCl_2_ (a chemical hypoxia mimic) can stabilize HIF-1alpha (encoded by *HIF-1A*) [38, 39], which is also elevated in FGR placentas [40, 41]. HIF-1 is a transcription factor that is specifically activated by hypoxia; it is a heterodimer composed of the HIF-1alpha and the HIF-1beta subunits [42]. HIF-1alpha is degraded under normoxic conditions, but rapidly accumulates in hypoxic conditions, where it can combine with HIF-1beta to transcriptionally modulate the expression of HIF-1 responsive downstream genes [42, 43]. CoCl_2_ treatment mimics a hypoxic microenvironment by inhibiting HIF-1alpha degradation, and 200 μM CoCl_2_ has effectively induced HIF-1alpha in cultured placental explants [27]. As expected, mRNA expression of *HIF-1A* was significantly increased in explants treated with CoCl_2_. We also observed increased immunostaining of HIF-1alpha protein in explants exposed to CoCl_2_ or 1% O_2_, but not in explants cultured in 21% O_2_. Although a previous study reported 200 μM CoCl_2_ effectively mimicked a hypoxic environment in the placental explants [27], to the best of our knowledge it is unclear whether 200 μM CoCl_2_ is equivalent to culture in 1% O_2_, which might explain the differences observed between the two culture conditions in this study. Kumar et al. showed high levels of HIF-1alpha protein expression can downregulate both ESRRG mRNA and protein expression in primary second-trimester cytotrophoblast cultured in 2% O_2_ [12]. The current study used an *in-vitro* placental explant model and observed similar findings following treatment with CoCl_2_ or culture in 1% O_2_, specifically, increased protein expression of HIF-1alpha which correlated with reduced expression of ESRRG expression.

Both treatments (CoCl_2_ and 1% O_2_) reduced hCG secretion and modestly increased LDH release, compared to control explants cultured at 21% O_2,_ suggesting these pathways might be regulated by the O_2_-sensitive transcription factor, HIF-1alpha. In common with previously reported findings in second trimester placental tissue, there was lower ESRRG expression at both the mRNA and protein level [12]. As ESRRG localizes to the syncytiotrophoblast layer and stromal cells in placental explants, one could hypothesize that it is involved in regulating trophoblast function and cell turnover. However, the levels of ESRRG protein measured by Western blotting after four days of culture were much lower than in fresh tissue and were not significantly decreased at 1% O_2_. This suggests that hypoxic culture may exert compensatory effects on the translation of the *ESRRG* pathway in the villous explant model, and there also could be other regulators of the signaling pathways involving *ESRRG* in hypoxia; to further explore this pathway, the identification of *ESRRG*’s downstream effectors is needed.

All of the genes downstream of *ESRRG* measured here were reduced following culture in 1% O_2_ or following treatment with CoCl_2_. Previously *CYP191.1* has been validated as a hypoxia-responsive downstream effector of *ESRRG* in primary second-trimester cytotrophoblast [12]. Our findings provide further evidence for the regulation of *CYP191.1* by *ESRRG*; however, to the best of our knowledge, our data are the first to suggest that *ESRRG* might mediate the expression of *HSD11B2* in hypoxia. The reduced expression of *HSD11B2* in hypoxia has been established previously in vitro and in vivo studies of pregnancy [44, 45]. Homan et al. found a low promoter activity in *HSD11B2* in a primary cytotrophoblast model under hypoxic conditions (1% O_2_) [18], suggesting that this may be in response to a lack of an upstream stimulus. As we report low mRNA expression of *HSD11B2* in our explant model in both 1% O_2_ and CoCl_2_ groups, it is possible that low levels of *HSD11B2* are mediated by hypoxia-induced reductions in the *ESRRG* signaling pathway. To determine whether these effects are HIF-dependent would require further experiments that reduce HIF-1alpha, for example, using siRNA, to determine whether the changes in *ESRRG* signaling persist.

### GSK5182 and DY131 rescue hypoxia-induced alterations in the *ESRRG* signaling pathway

In this study, GSK5182 and DY131 rescued the reduction in *ESRRG* expression, and its downstream genes induced by hypoxia. Interestingly, we observed that GSK5182, an inverse agonist for *ESRRG*, can induce ESRRG protein expression, as well as that of its downstream genes, in hypoxia. In previous studies using cell lines, GSK5182 was shown to inhibit the transcription of *ESRRG* [46, 47]. However, the cultured villous explant model in the current study had very low protein expression of *ESRRG*. Therefore, it is possible that GSK5182 could not further reduce the gene expression of *ESRRG,* but instead induced its expression in hypoxia by activating coactivators or other regulators of *ESRRG*. Further studies are needed to uncover this unexpected effect of GSK5182 in placental villous explants.

DY131, an agonist of *ESRRG*, can induce the expression of *ESRRG* in animal models or primary cytotrophoblast [13, 22, 23, 48]. Our study suggests that DY131 not only increases the mRNA expression of *ESRRG* and its downstream genes (*HSD11B2* and *CYP191.1*) in 21% O_2,_ but can also restore protein expression of ESRRG and its downstream genes (*HSD11B2* and *CYP191.1*) in hypoxia. This further supports that both *HSD11B2* and *CYP191.1* are downstream genes of *ESRRG* in the placenta. This restoration of effect supports the hypothesis that *ESRRG* can regulate some of the effects of hypoxia in trophoblast, by mediating the expression of its downstream genes, *HSD11B2* and *CYP191.1*.

### Potential therapeutic efficacy of the *ESRRG*’s agonist DY131 in the placental dysfunction

The reduction in hCG secretion on day 4 of culture in 1% O_2_ is consistent with previous reports of this explant model or primary trophoblast [9, 37, 49, 50], which suggests an impaired differentiation of syncytiotrophoblast in hypoxia. Like our previous study [9], hypoxia reduced the number of cells in cycle and increased apoptosis. DY131 treatment reduced the level of cell necrosis, increased the number of cells in cycle, and reduced apoptosis in explants cultured under hypoxic conditions, but did not have any effect on hCG. These results suggest that *ESRRG* signaling mediates some of the downstream effects of hypoxia, which are implicated in the pathophysiology of FGR. Two studies have already identified the possibility of using DY131 as a therapeutic intervention in mice [13, 51], and our observations suggest that DY131 could be investigated as a potential therapeutic agent to treat hypoxia-induced placental dysfunction. However, further studies are required to assess the safety and efficacy of DY131 in pregnant animal models by targeting ESRRG signaling in specific pregnancy complications, such as FGR. To avoid problems with reduced efficacy and off-target effects in other organs that express ESRRG, approaches that facilitate placental-specific delivery of DY131 should be considered [52, 53].

### Strengths and limitations

This study has further confirmed that *ESRRG* signaling is reduced in placentas from FGR pregnancies, by applying a more stringent definition of FGR in a population not previously studied. Unlike the previous study [12], we firstly explored the relationship between hypoxia-mediated *ESRRG* signaling pathways and cell turnover in a cultured third-trimester villous explant model, which may be more physiologically relevant than isolated second-trimester primary cytotrophoblast, as cell–cell interactions between stromal cells, cytotrophoblast and syncytiotrophoblast are maintained. In addition, this is the first study to explore whether *ESRRG*’s agonist, DY131, can restore impaired *ESRRG* signaling and rescue the aberrant cell turnover observed in villous explants exposed to hypoxia. But whether DY131 could target the placental dysfunction underlying FGR placentas by mediating *ESRRG* signals would be further investigated in the future.

Although our results are consistent with previous reports in other populations, further studies are needed to confirm whether *ESRRG* is reduced in other populations with FGR and whether there is a relationship with disease severity. To explore whether *ESRRG* mediated the placental phenotype seen in FGR, this study used a short-term culture of placental villous explants, but the time period selected was appropriate to study the effects of longer-term culture, which should also be explored.

## Conclusion

This study shows that *ESRRG* signaling is dysregulated in FGR. The molecular mechanism underlying the regulatory role of *ESRRG* in FGR appears to be mediated in part through the hypoxia-sensitive *ESRRG* signaling pathway. An *ESRRG* agonist, DY131, can increase *ESRRG* signaling and rescue the abnormal cell turnover observed in placental explants cultured under hypoxia. Modulation of this signaling pathway offers a novel therapeutic option for the treatment of FGR.

## Data availability

The data underlying this article will be shared on reasonable request to the corresponding author.

## Conflict of interest

The authors have declared that no commercial or financial conflict of interest exists.

## Authors’ contribution statement

Z.Z., L.K.H., K.F., and A.E.P.H. conceived and designed the research. Z.Z. conducted the experiments. A.E.P.H., K.F., and L.K.H. contributed to the reagents and analytical kits, and Z.Z. analyzed and interpreted the data with assistance from the other authors, and drafted the manuscript. All authors read and approved the final manuscript.

## Supplementary Material

Zou_SupF_1_ioac108

Zou_SupF_2_ioac108

ZOU_SupF_3_ioac108

ZOU_SupF_4_ioac108

ZOU_SupF_5_ioac108

ZOU_SupF_6_ioac108

Zou_SupF_7_ioac108

ZOU_SupF_8_ioac108

Supplementary_Table_1_BOR_ioac108
