# Supplementary figures and images for: Placental expression of estrogen-related receptor gamma is reduced in fetal growth restriction pregnancies and is mediated by hypoxia
Source: Biol Reprod. 2022 May 19;107(3):846–57. doi: 10.1093/biolre/ioac108 (PMC9476228; doi:10.1093/biolre/ioac108)

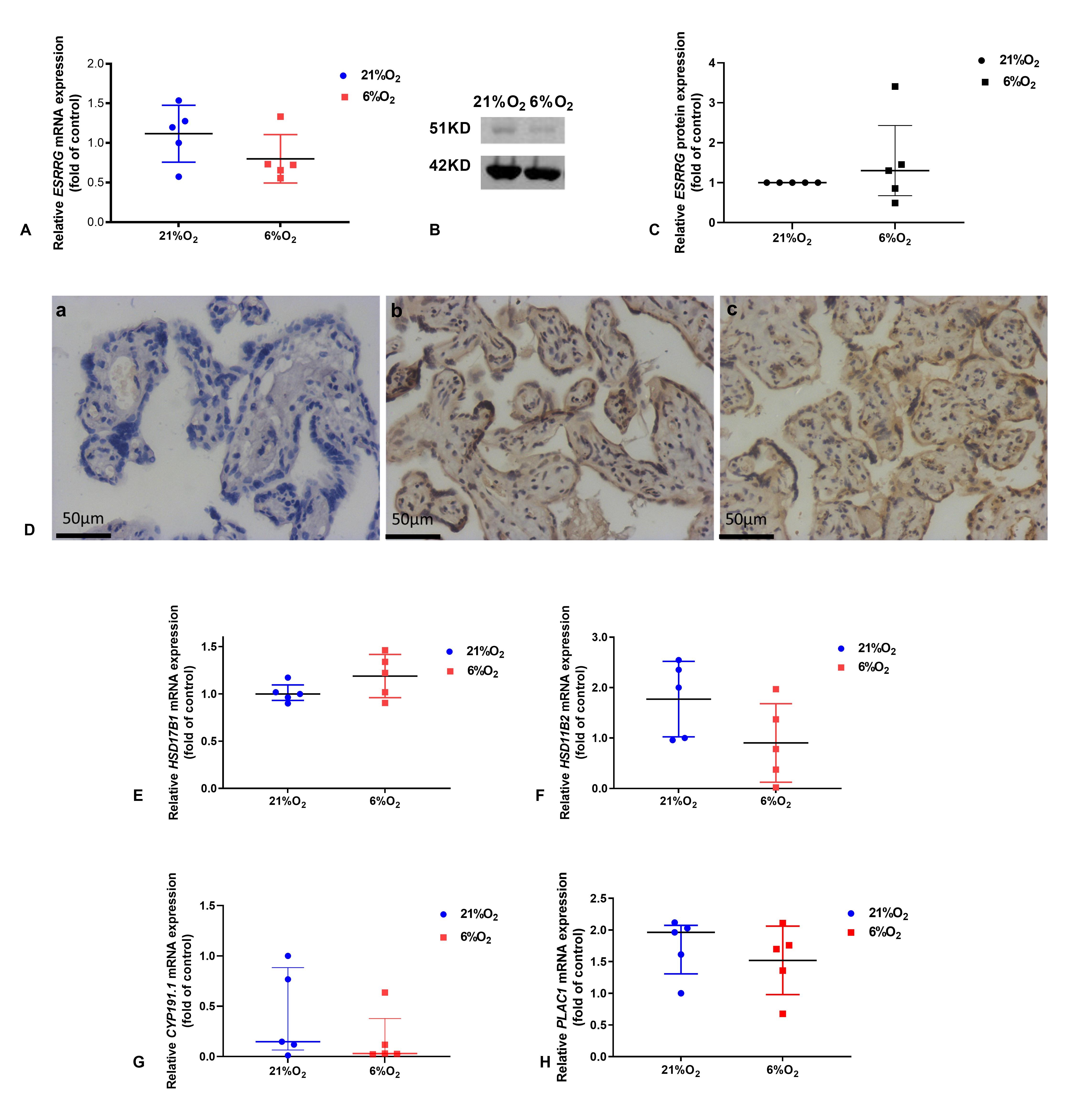

Supplement: Zou_SupF_1_ioac108 [file zou_supf_1_ioac108.jpeg]

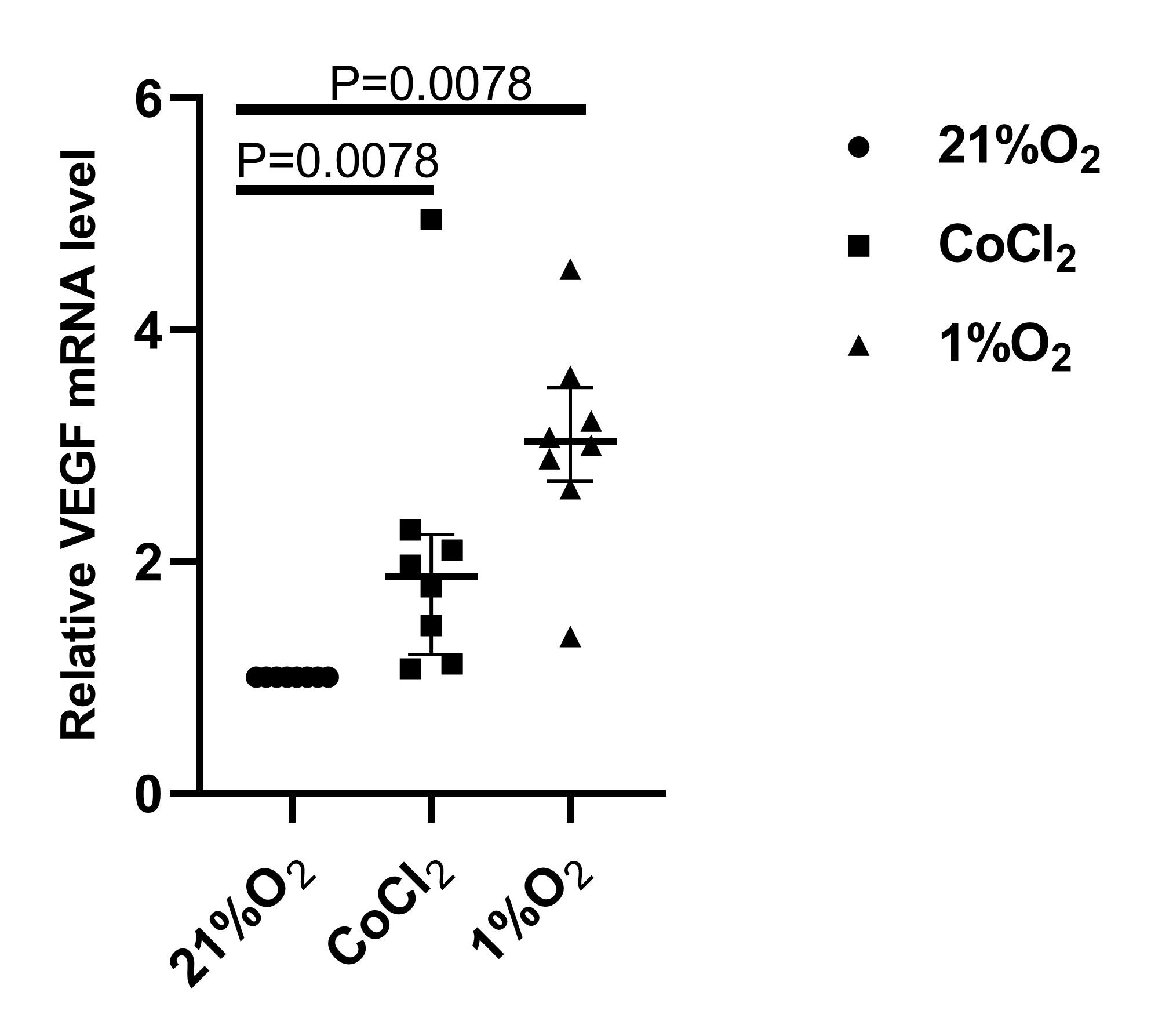

Supplement: Zou_SupF_2_ioac108 [file zou_supf_2_ioac108.jpeg]

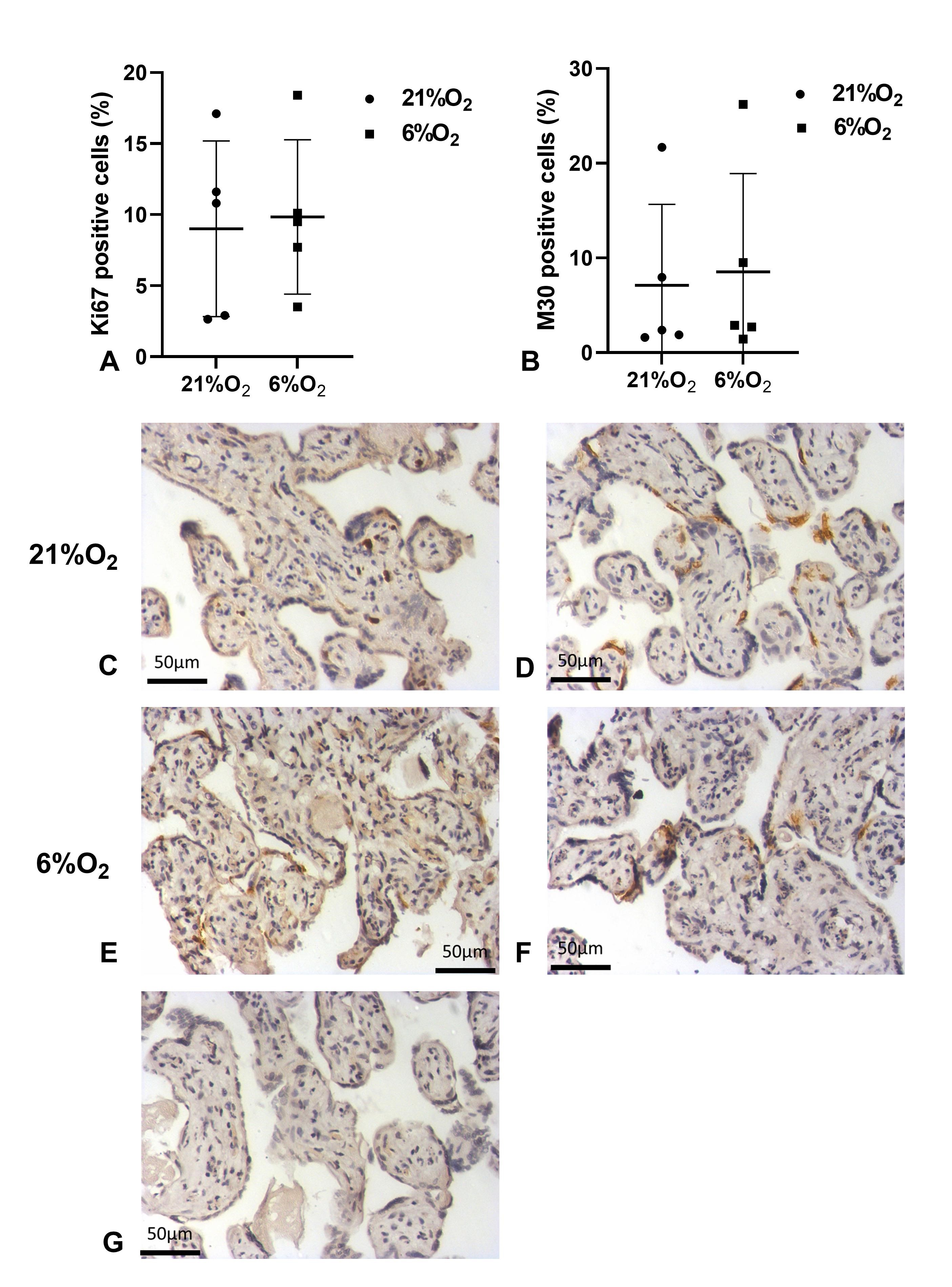

Supplement: ZOU_SupF_3_ioac108 [file zou_supf_3_ioac108.jpeg]

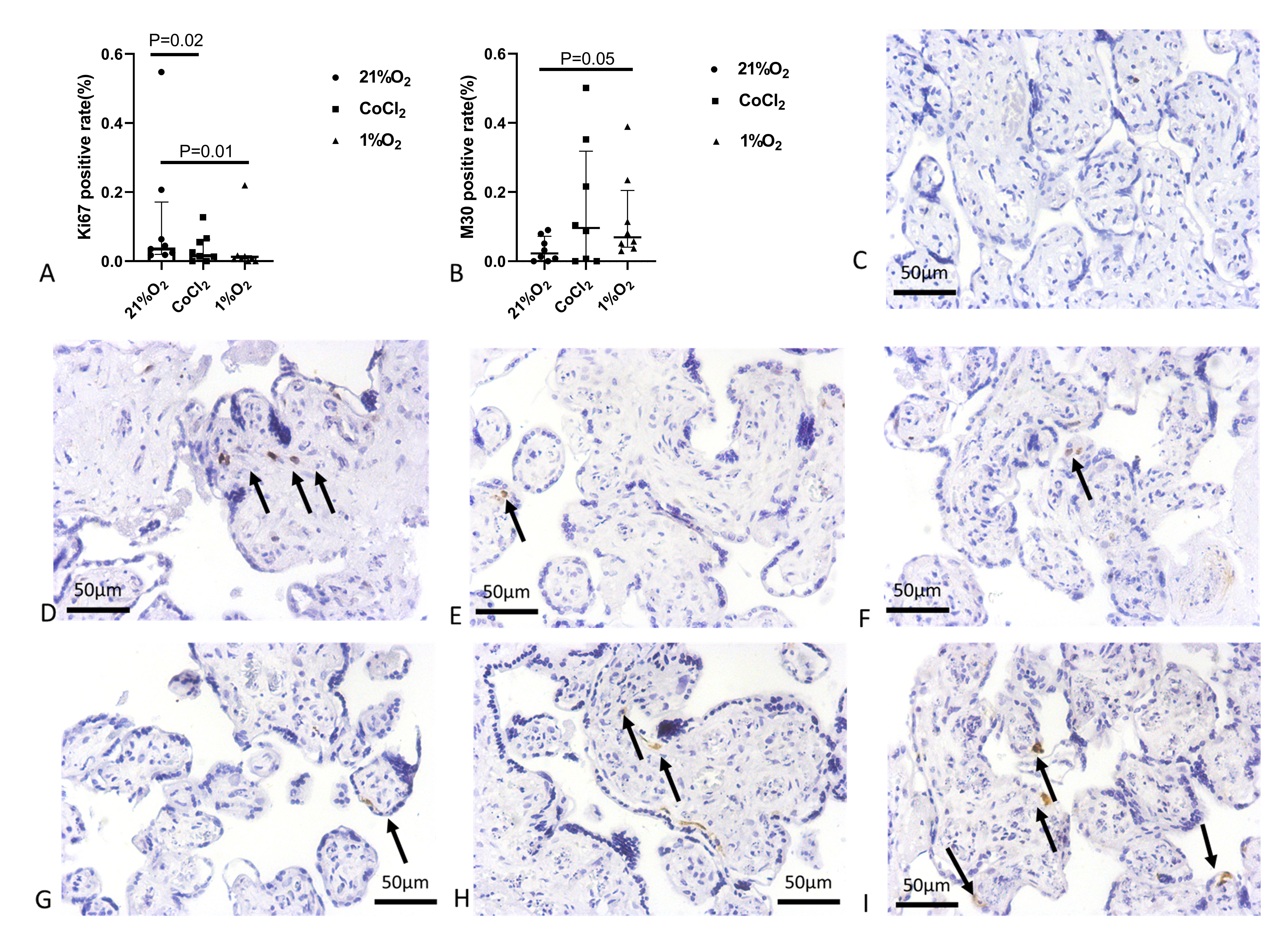

Supplement: ZOU_SupF_4_ioac108 [file zou_supf_4_ioac108.jpeg]

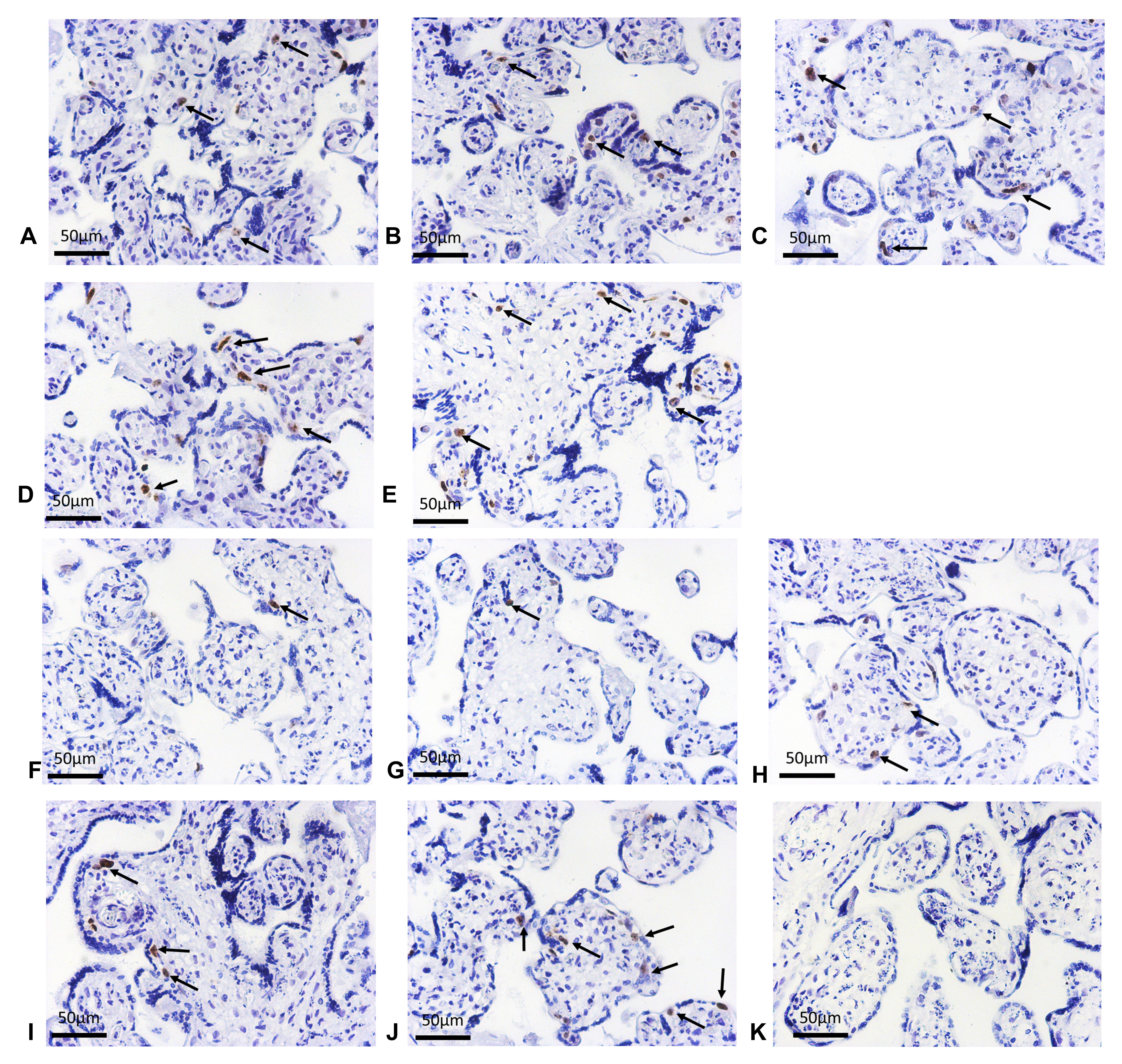

Supplement: ZOU_SupF_5_ioac108 [file zou_supf_5_ioac108.jpeg]

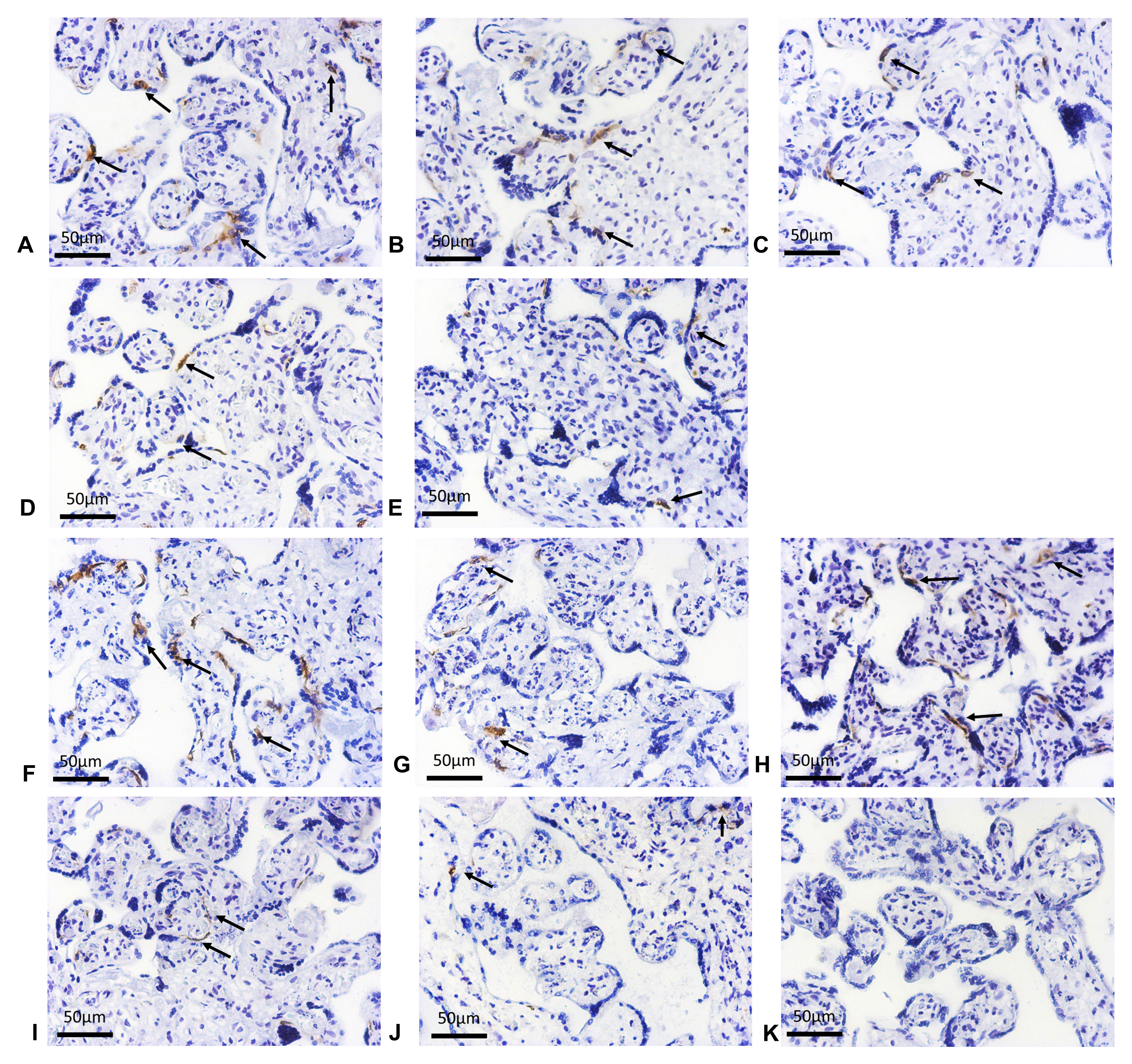

Supplement: ZOU_SupF_6_ioac108 [file zou_supf_6_ioac108.jpeg]

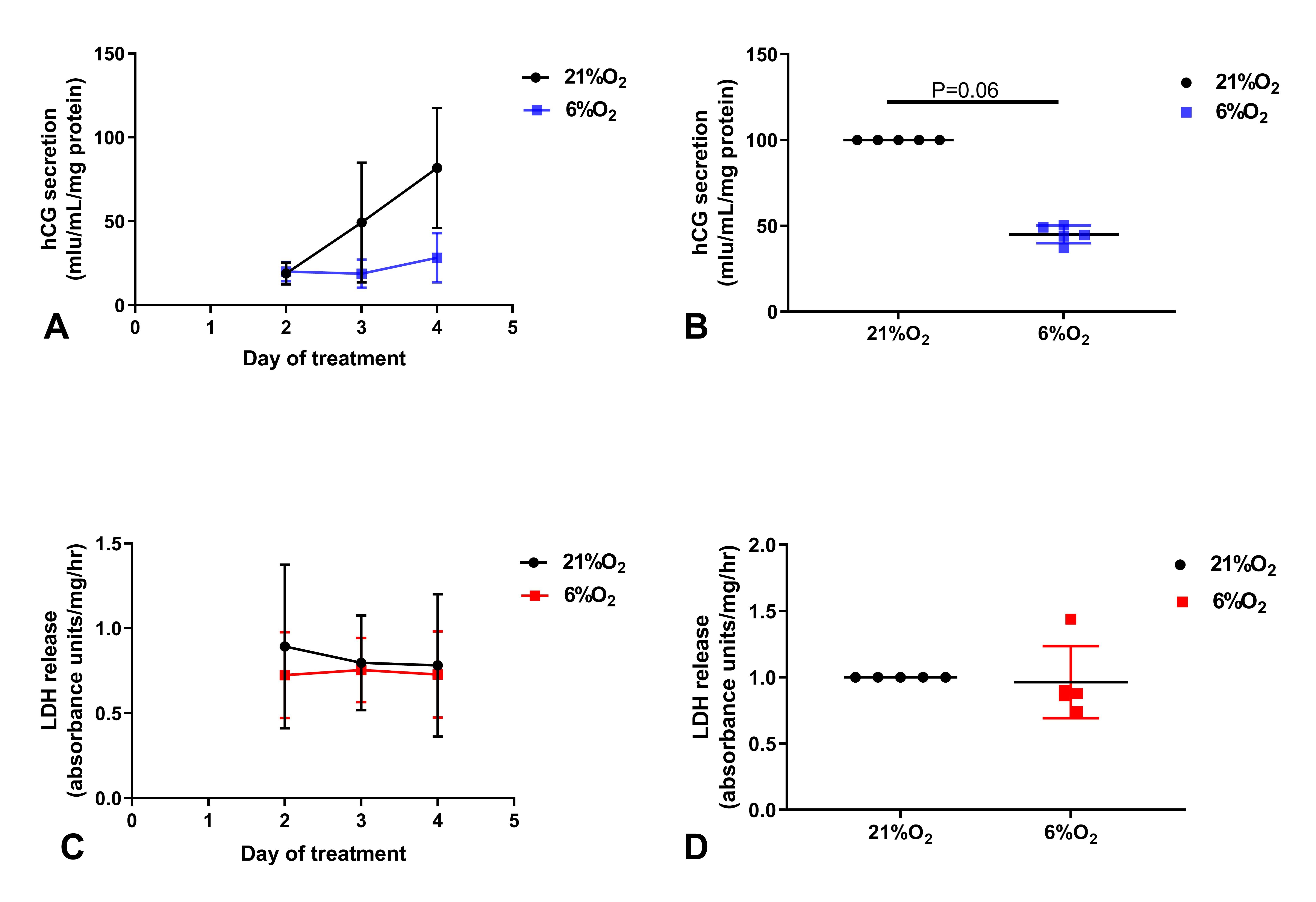

Supplement: Zou_SupF_7_ioac108 [file zou_supf_7_ioac108.jpeg]

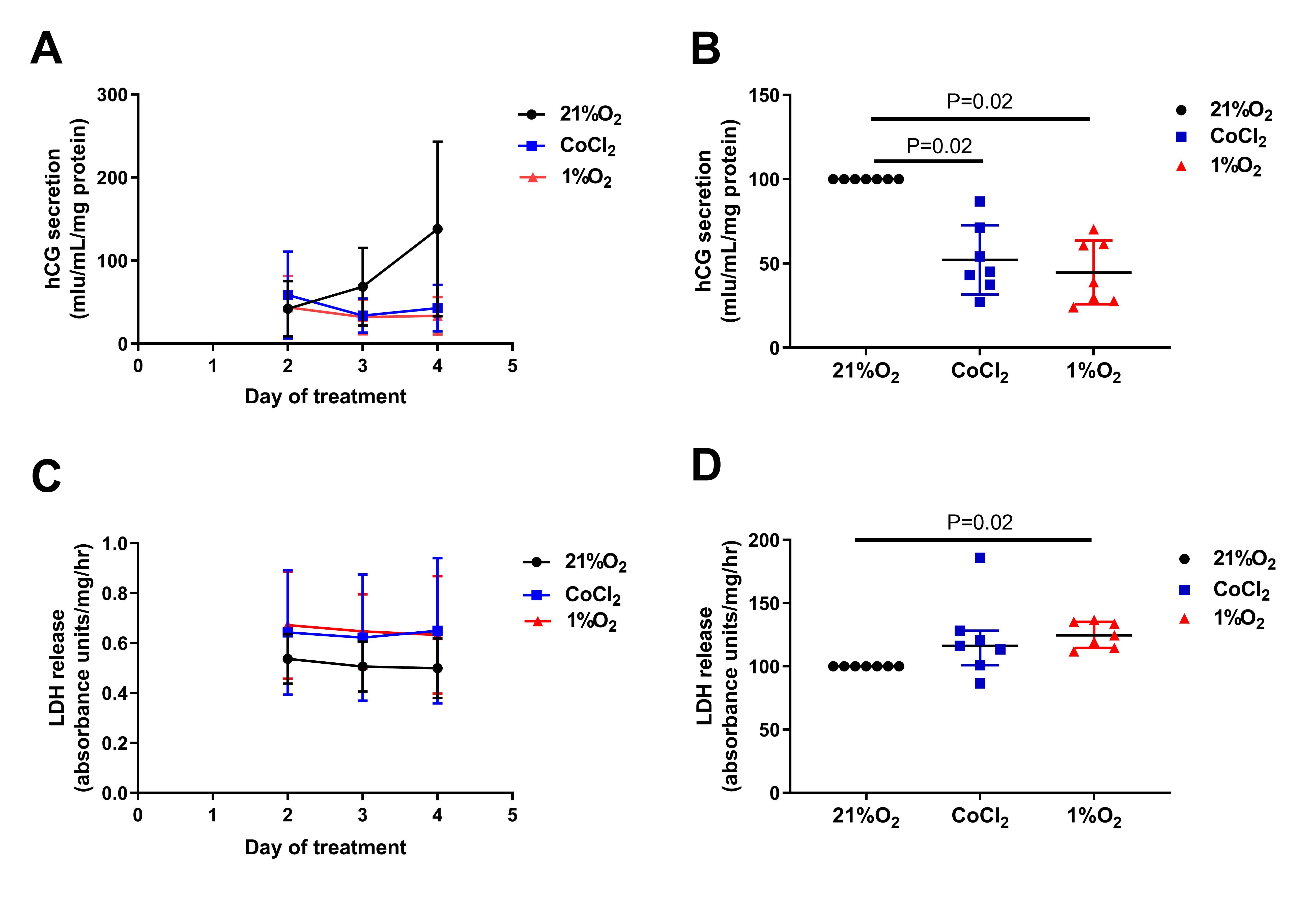

Supplement: ZOU_SupF_8_ioac108 [file zou_supf_8_ioac108.jpeg]
