# Supplementary material for: Placental expression of estrogen-related receptor gamma is reduced in fetal growth restriction pregnancies and is mediated by hypoxia
Source: Biol Reprod. 2022 May 19;107(3):846–57. doi: 10.1093/biolre/ioac108 (PMC9476228; doi:10.1093/biolre/ioac108)
Supplement: Supplementary_Table_1_BOR_ioac108 [file supplementary_table_1_bor_ioac108.docx]

| Primer set | Sequence |
| --- | --- |
| *HIF-1A* | Forward: 5′-GCCGCTGGAGACACACAATCAT-3  Reverse: 5′-TCCATCGGAAGGACTAGGTGT-3 |
| *VEGF* | Forward: 5′-CTACCTCCACCATGCCAAGT-3  Reverse: 5′-GCAGTAGCTGCGCTGATAGA-3 |
| *ESRRG* | Forward: 5’-CTG ACG GAC AGC GTC AAC C-3’  Reverse: 5’-GGC GAG TCA AGT CCG TTC TG-3’ |
| *CYP191.1* | Forward: 5’-ACG GAA GGT CCT GTG CTC G-3’  Reverse: 5’-GTA TCG GGT TCA GCA TTT CCA-3’ |
| *HSD11B2* | Forward: 5’-GAC CTG ACC AAA CCA GGA GA-3’  Reverse: 5’-GCC AAA GAA ATT CAC CTC CA-3 |
| *HSD17B1* | Forward: 5’-GCC TTC ATG GAG AAG GTG TT-3’  Reverse: 5’-CGA AAG ACT TGC TTG CTG TG-3’ |
| *PLAC1* | Forward: 5’-ATT GGC TGC AGG GAT GAA AG-3’  Reverse: 5’-TGC ACT GTG ACC ATG AAC CA-3’ |
| *RPLP0* | Forward: 5’-TGC ATC AGT ACC CCA TTC TAT CA-3’  Reverse: 5’-AAG GTG TAA TCC GTC TCC ACA GA-3’ |

Supplementary Table 1. Primer sequences for *HIF-1A*, *ESRRG*, its downstream genes and *RPLP0*.
